# Crystallographic structure of ubiquitin in complex with cadmium ions

**DOI:** 10.1186/1756-0500-2-251

**Published:** 2009-12-15

**Authors:** Insaf A Qureshi, Francois Ferron, Cheah Chen Seh, Peter Cheung, Julien Lescar

**Affiliations:** 1 School of Biological Sciences, Nanyang Technological University, 60 Nanyang Drive, 637551, Singapore; 2 AFMB UMR 6098 CNRS, Marseille, France

## Abstract

**Background:**

Ubiquitination plays a critical role in regulating many cellular processes, from DNA repair and gene transcription to cell cycle and apoptosis. It is catalyzed by a specific enzymatic cascade ultimately leading to the conjugation of ubiquitin to lysine residues of the target protein that can be the ubiquitin molecule itself and to the formation of poly-ubiquitin chains.

**Findings:**

We present the crystal structure at 3.0 Å resolution of bovine ubiquitin crystallized in presence of cadmium ions. Two molecules of ubiquitin are present in the asymmetric unit. Interestingly this non-covalent dimeric arrangement brings Lys-6 and Lys-63 of each crystallographically-independent monomer in close contact with the C-terminal ends of the other monomer. Residues Leu-8, Ile-44 and Val-70 that form a hydrophobic patch at the surface of the Ub monomer are trapped at the dimer interface.

**Conclusions:**

The structural basis for signalling by poly-Ub chains relies on a visualization of conformations of alternatively linked poly-Ub chains. This arrangement of ubiquitin could illustrate how linkages involving Lys-6 or Lys-63 of ubiquitin are produced in the cell. It also details how ubiquitin molecules can specifically chelate cadmium ions.

## Background

Ubiquitin (Ub) is an evolutionary conserved protein of 76 amino-acids found in all eukaryotes. Ub participates in the regulation of various cellular processes through conjugation to other cellular proteins, which is catalyzed by an enzymatic cascade that involves the sequential actions of three enzymes known as Ub-activating (E1), Ub-conjugating (E2) and Ub-protein ligase (E3) enzymes. As a result, the C terminal glycine residue of Ub is ligated to the target protein through the formation of an isopeptide bond to one of its lysine residues, by a complex formed by the E2 and E3 enzymes. Covalent attachment of the C-terminal end of Ub to lysine residues of a protein substrate, a process known as ubiquitination, targets the substrate for a range of possible fates including protein degradation. Conjugation of a single Ub molecule regulates transcription, endocytosis and membrane trafficking. Since the ubiquitin protein itself contains seven lysine residues: Lys-6, Lys-11, Lys-27, Lys-29, Lys-33, Lys-48 and Lys-63, additional Ub molecules can be ligated to each of these seven sites, leading to the formation of poly-Ub chains with distinct linkages between individual Ub molecules. The most common poly-Ub chain is linked through Lys-48 and serves as a signal for rapid degradation of substrates by the proteasome-dependent proteolysis pathway [1]. Poly-Ub chains linked through Lys-63 trigger non-proteolytic signals, such as for DNA repair, ribosomal protein synthesis, inflammatory signalling, endocytosis and vesicular trafficking. However, a recent report suggested that *in vivo*, Lys-63-linked ubiquitin chain may also serve as a targeting signal for the 26S proteaseome [2]. Lys-6-modified ubiquitin is a potent and specific inhibitor of ubiquitin-mediated protein degradation [3]. Lys-11- and Lys-29-linked chains also target the substrate for proteasome-dependent protein degradation. Lys-29- or Lys-33-linked ubiquitin chains regulate the enzymatic activity of kinases [4]. Thus, different poly-Ub chains, despite being assembled from identical Ub units are recognized as distinct signals by the cell machinery. Therefore, a visualization of conformations of alternatively linked poly-Ub chains should help deciphering the molecular and structural basis for their signalling activities.

In the present study, we crystallized bovine ubiquitin in the presence of a high concentration of cadmium salt in a form that comprises two independent molecules per asymmetric unit and report this structure at 3Å resolution.

## Methods

### Crystallization

Bovine ubiquitin (Sigma) was subjected to gel filtration chromatography on a HiLoad 16/60 Superdex 75 column (GE Healthcare) using buffer A (50 mM HEPES pH 7.5, 150 mM NaCl and 5 mM dithiothreitol). Fractions were pooled and concentrated to 10 mg/ml in buffer A using an Amicon Ultra-5 filter (Millipore). Screening for crystallization conditions was performed using sitting-drop vapour diffusion in 96-well plates by mixing a volume of 0.2 μl of protein solution with 0.2 μl of precipitant. Drops were equilibrated against 0.1 ml of precipitant solution at 291 K. Crystallization screens tested included the sparse-matrix Crystal Screens 1 and 2, Crystal screen Lite & Cryo, Index, SaltRx, MembFac, PEG/Ions, grid screens for sodium malonate and ammonium sulfate (Hampton Research) using a CyBio crystallization robot. Crystals were observed using a reservoir solution containing 100 mM HEPES pH 7.5, 50 mM cadmium sulfate, 1 M sodium citrate.

### Data collection and structure determination

Crystals suitable for diffraction experiments grew within one week to maximum dimensions of approximately 0.20 × 0.25 × 0.25 mm and diffracted to 3.0 Å resolution at third generation synchrotron beamlines. The data set was collected at beamline ID14-4, ESRF (Grenoble, France) using crystals frozen in the reservoir solution supplemented with 30% glycerol. Reflection data were indexed, integrated and scaled using MOSFLM and the CCP4 program suite (Table 1). The structure was determined by molecular replacement with the program MOLREP [5] using the monomeric Ub structure (PDB ID code 1UBQ) as a search probe. The model was refined using the program CNS [6] and rebuilt with program O [7]. The geometry of the final model was checked with PROCHECK [8] and figures for molecular representation were prepared using PyMOL http://www.pymol.org. Statistics for structure refinement and validation are given in Table 1. The coordinates are available from the Protein Data Bank with accession number 3H1U.

**Table 1:** Data collection and refinement statistics

*Data collection statistics*	
Space group	P4(3)32

Unit cell a, b, c (Å)	105.23

No. of molecules per asymmetric unit	2

Resolution (Å)	30.0-3.0

No. of measured reflections	92197

No. of unique reflections	4367

Completeness	99.9 (100.0)

Redundancy	21.1 (22.9)

R_sym _(%)	6.9 (56.7)

*I/σ(I)*	35.7 (5.8)

*Refinement statistics*	

No. of reflections (working set/test set)	3613/172

R factor (R_work_/R_free_)	0.222/0.257

No. of atoms	

Protein	1196

Cadmium	5

Water	42

B mean (Å^2^)	87.88

B (Wilson Plot, Å^2^)	101.29

B Protein atoms (Å^2^)	72.37

B cadmium atoms (Å^2^)	69.76

B water atoms (Å^2^)	59.02

*R.M.S. deviations*	

Bond lengths (Å)	0.006

Bond angles (°)	1.44

*Ramachandran statistics (%)*	

Most favored	81.8

Additionally allowed	17.4

Generously allowed	0.8

Disallowed	0.0

*Values in parentheses refer to the corresponding values of the highest resolution shell (3.00-3.12)

## Results and Discussion

### Overall Structure

**Figure 1:**
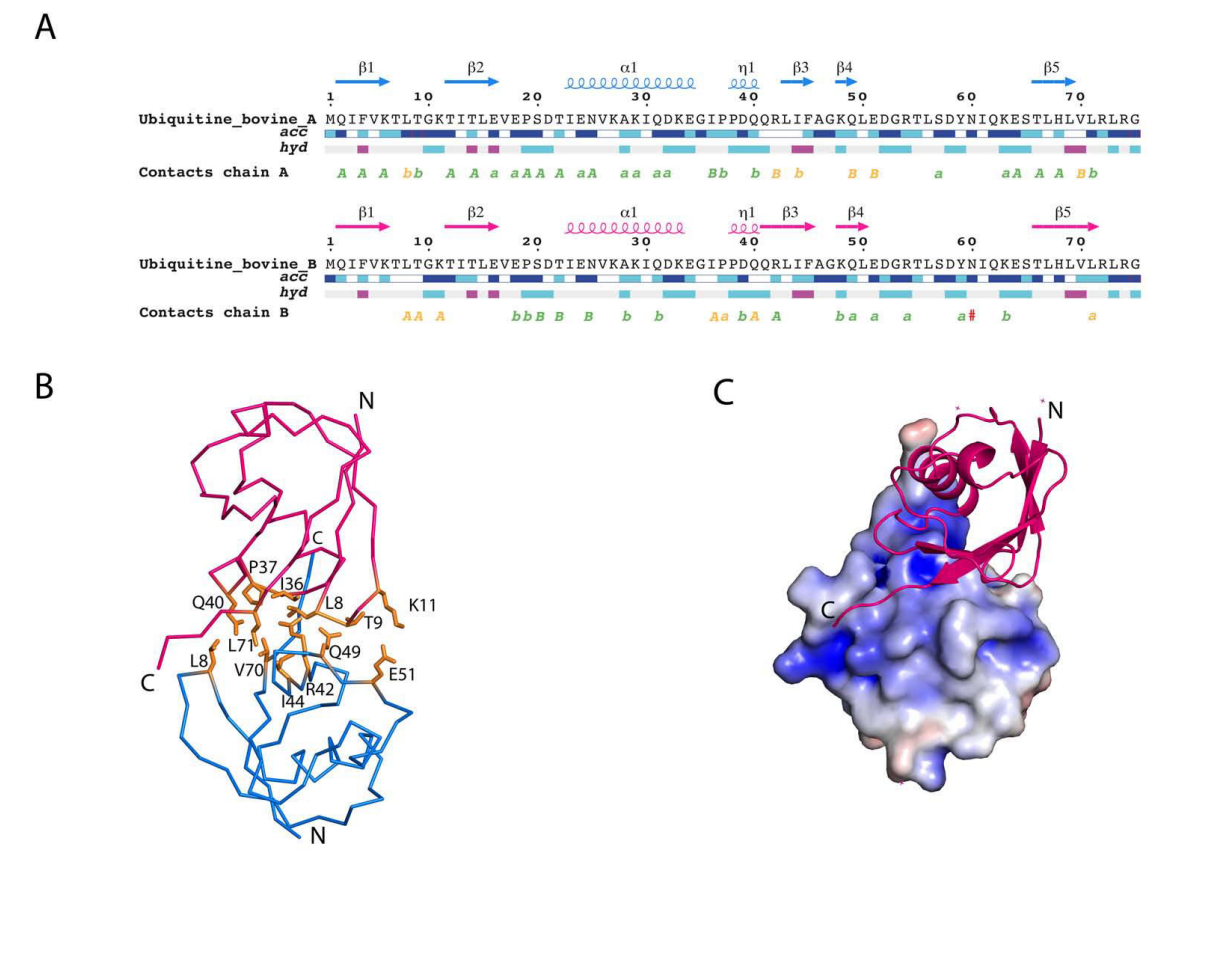
**Overall Structure**. A. Schematic representation of the intermolecular contacts established by Ub monomer A (blue) and B (pink). Non-crystallographic (yellow) or crystallographic (green) contacts are marked under the corresponding residue. A capital letter marks an interaction <3.2Å and a small letter a contact <4Å. B. Secondary structure elements are indicated above the respective sequence. The relative accessibility (acc) is calculated by DSSP and rendered as blue-colored boxes (from dark-blue: fully accessible, to white: buried). The hydropathic character of a sequence is calculated according to the algorithm of Kyte and Doolittle with a window of 3 and is rendered as red (hydrophobic) to blue (hydrophilic) boxes. C. The Ub non-covalent crystallographic dimer using the color scheme in A). Side-chains of residues involved in the association are represented as yellow sticks and labeled. N- and C-terminal ends of each Ub chain are indicated.

**Figure 2:**
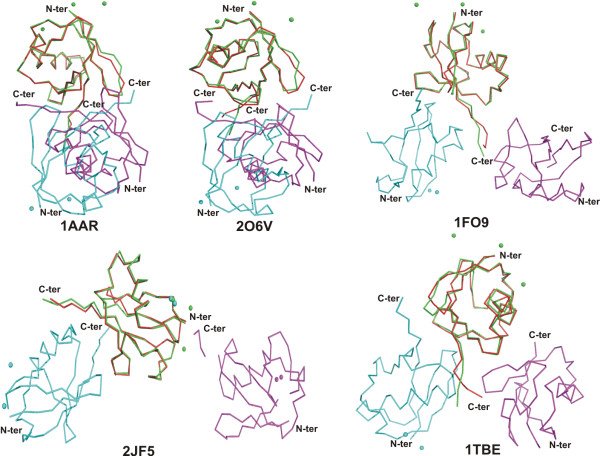
**Comparison of Ub crystallographic dimers**. Monomer A of Ub (in green, this work) was superimposed with one monomer of either 1AAR, 2O6V, 1F09, 2JF5, 1TBE. Only the α-carbon traces are displayed. The monomer B of Ub is in cyan, while the A and B monomers of other ubiquitins are colored in red and magenta. When present, cadmium ions from the crystallization media are shown as spheres.

**Figure 3:**
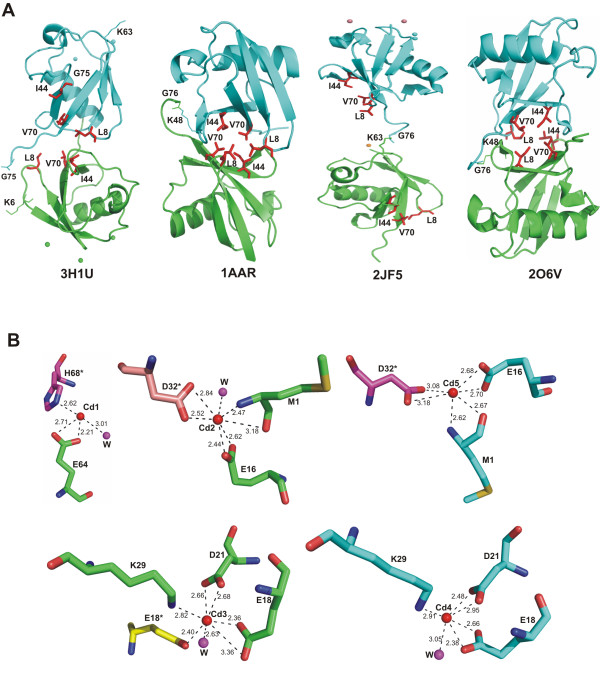
**The hydrophobic patch and binding of cadmium ions**. A. Mapping the locations of the residues that form the hydrophobic patch (Leu-8, Ile-44, and Val-70) with respect to the crystallographic dimer interface in four Ub crystal structures. Chain A are shown in green and chain B in cyan. Residues from the hydrophobic patch are shown as red sticks and labelled. When present, cadmium ions are shown as either green or cyan spheres, according to the Ub monomer they bind to. B. The five cadmium binding sites observed. Residues of chain A are in green while symmetry related molecules are shown in different colours. Contacts between cadmium ions (red spheres) and water molecules (magenta spheres) are indicated by dashed lines, with the corresponding distances labelled in Å. Oxygen atoms are coloured in red and nitrogen atoms in blue.

The asymmetric unit contains two non-covalently linked Ub monomers (Figure. 1) hereafter named A and B that can be superimposed with an r.m.s. deviation of 0.89 Å for 75 equivalent α-carbon atoms. The absence of covalent bond formation between the two Ub monomers was confirmed both by SDS PAGE and mass spectrometry using dissolved Ub crystals that only revealed the presence of monomeric species. Structural differences with other crystallized ubiquitins [9-11] are located in one loop spanning residues Thr-7-Gly-10 and at the C-terminal ends that are engaged in different crystal packing interactions (Figure. 2). Compared to the Lys-63-linked diubiquitin that was also crystallized in presence of cadmium ions [[11], PDB code 2JF5], significant variations in conformation are observed for monomer A with a r.m.s.d. of 0.88 and a r.m.s.d of 1.34 Å for the B monomer: Of note, the α-carbon atoms of residues Leu-8 and Thr-9 are displaced by 3.6 and 3.3 Å respectively compared to the Lys-63-linked diubiquitin structure. Residue Leu-8 is an important recognition element for the binding of Lys-48-linked tetraubiquitin to the 26S proteasome and the Leu-8Ala mutation causes the largest defect in substrate degradation as compared to other mutations targeting the hydrophobic patch (e.g. Ile-44Ala and Val-70Ala) [12,13]. The ubiquitin molecule displays extensive acidic and basic patches at opposite faces of the monomer. The hydrophobic patch at the surface of each ubiquitin molecule includes Leu-8, Ile-44 and Val-70 and mediates interaction between mono- and polyubiquitin and a variety of ubiquitin-binding domains [14]. In the present crystal structure these residues are trapped at the interface between monomers A and B (Figure 3A). A list of the interactions between the two crystallographically-independent Ub monomers is given in Table 2. Interestingly, the relative arrangement of the two Ub monomers brings Lys-6 from monomer A in close proximity to the C-terminal end of monomer B. Likewise, Lys-B63 is next to the C-terminus of a symmetry-related molecule of chain A (Figure 3A). Recently, it was shown that formation of Lys6-linked polyubiquitin chains was catalysed by a heterodimeric RING E3 ligase complex consisting of the breast cancer-susceptibility protein BRCA1 and BARD1 [15]. As BRCA1/BARD1 are localized at sites of DNA damage through binding of their adaptor protein RAP80 to Lys6- and Lys63-linked ubiquitin chains [16], both of these chain types might be involved in DNA repair.

**Table 2:** Non-covalent interactions between two ubiquitin monomers

Molecule A		Molecule B		
	
Residue	Atom	Residue	Atom	Distance (Å)
Leu8	CD1	Gln40	NE2	3.23

Leu8	CD2	Gln40	OE1	3.99

Gln40	N	Leu8	O	2.70

Gln40	OE1	Thr9	CG2	3.73

Gln40	NE2	Thr9	CG2	2.91

Arg42	NH1	Glu34	O	2.69

Arg42	NH1	Ile36	N	3.17

Arg42	NH2	Pro37	CD	3.38

Ile44	CG2	Leu71	CD2	3.44

Glu51	OE2	Lys11	NZ	3.19

Val70	CG1	Gln40	OE1	3.20

Val70	CG2	Gln40	NE2	3.69

The buried surface area between the two monomers present in the asymmetric unit is 1082 Å^2 ^indicating that this dimeric arrangement will not be maintained in solution. This was confirmed by gel filtration experiments (not shown). Interestingly, the mode of association in the crystal between molecules A and B (Figure 3A) is grossly related to the Lys-48-linked diubiquitin structure observed by Cook et al [9] which also buries the hydrophobic residues (Leu-8, Ile-44 and Val-70) at the interface but has a much more extensive dimerization interface of 1507 Å^2 ^([9] PDB code 1AAR]. However, no clear correlation seems to exist between isopeptide bond formation, the oligomerization state and the surface area buried at the molecular interface: tetra-ubiquitin chains have been observed in various crystal structures either with a surface of 1492 Å^2 ^for the Lys-48-linked tetra-ubiquitin ([17] PDB code 2O6V] or with much smaller buried interface of 659 Å^2 ^([10] PDB code 1TBE], 435 Å^2 ^([18] PDB code 1F9J], or 209 Å^2 ^([11] PDB code 2JF5]. The Lys-63-linked diubiquitin ([11] PDB code 2JF5] adopts an extended conformation with no clear interface formation between Ub domains and fully exposes both hydrophobic patch residues of each Ub to the solvent. Thus, in the present structure, the overall conformation of ubiquitin molecules differs from those seen in previously reported poly-Ub structures (Figure 3A), which probably accounts for distinct roles in cell signalling. The analysis of crystal packing shows that both molecules within the asymmetric unit come in close contact with symmetry-related molecules (Additional File 1: Fig. S1). An electron density omit map after removing Gly-75 of both chains from the phasing model, indicates that these residues are well ordered in the crystal (Additional File 2: Fig. S2). Although all known Ub structures are closely related but their modes of association differs. Thus the identification of a new interface may explain the formation of Lys-6 or Lys-63 linked diubiquitin.

### Metal binding

Molecule A interacts with three cadmium ions and molecule B chelates two cadmium ions. Cadmium ions contribute directly to lattice formation by bridging residues from four neighbouring ubiquitin molecules. The first cadmium ion (Cd1) completes its tetrahedral coordination sphere by binding the carboxylic group of Glu64, atom NE2 of His68* (the star denotes a residue from a symmetry-related protein) and one water molecule (Figure 3B). Cadmium ions Cd2 and Cd5 are bound to the carboxylic group of Glu16, main chain amide and carbonyl groups of residue Met1 and the carboxylic group of Asp32* and a water molecule. Cd2 binds to a water molecule but Cd5 does not interact with any water molecule (Figure 3B). Cadmium ions Cd3 and Cd4 are chelated by the carboxylic groups of Glu18 and Asp21 and atom NZ of Lys29. One water molecule completes their coordination shells (Figure 3B). Interestingly, residues that bind cadmium ions, especially Glu16, Glu18 and Glu64 show a remarkable displacement from their positions in native ubiquitin; detailed views of the various cadmium binding sites are shown in Additional File 3: Fig. S3. A comparison of the crystal structure reported here with other ubiquitin complexed to metal ions ([11] PDB code 2JF5) is shown in Figure 2. In addition, three out of five cadmium ions bind to ubiquitin in different ways: Positions of Cd3 and Cd4 are analogous to that observed in structure 2JF5, but positions of Cd2 and Cd5 are occupied by magnesium and cobalt ions, respectively. No ligand is present in structure 2JF5 corresponding to the position occupied by Cd1. These results support the findings by Dokmanic and co-workers [19] stating that the most abundant amino-acid residues in the tetrahedral or octahedral cadmium coordination spheres are Cys followed by Glu and Asp.

## Competing interests

The authors declare that they have no competing interests.

## Authors' contributions

IAQ crystallized the protein and took part in refinement. FF collected diffraction data at ESRF. JL solved and refined the structure. All authors contributed reagents and participated in writing the paper.

## Supplementary Material

Additional file 1 **Figure S1: Packing of molecules in crystal of Ub**. A) The non-crystallographic dimer using a ribbon representation. B) Nearest symmetry molecules (at 4Å) in the same orientation as A). C, D, E) rotation of B) by 90° around the horizontal axis, at 90° along Y, at 180° along X respectively. F) rotation of C) at 180° along Y. Click here for file

Additional file 2 **Figure S2: Omit electron density map around the C-terminal of Ub**. Omit electron density map around the C-terminal of chain A (panel A) and chain B (panel B) after removing residue Gly75 from the phase calculation. The *F*_o _-*F*_c _difference electron density map contoured at 3.0 σ level is coloured in green and 2*F*_o _-*F*_c _map contoured at 1.5 σ level (in blue). Density accounting for Gly-75 is visible for both chains. Click here for file

Additional file 3 **Figure S3: Comparison between structures of native and cadmium bound Ub**. Superposition of the structures of native ubiquitin (magenta) and bovine ubiquitin (green) in the region of the binding sites of cadmium (red). The interactions between cadmium ions and donor atoms are indicated by dotted lines. Click here for file
