# Supplementary figures and images for: Crystallographic structure of ubiquitin in complex with cadmium ions
Source: BMC Res Notes. 2009 Dec 15;2:251. doi: 10.1186/1756-0500-2-251 (PMC2804574; doi:10.1186/1756-0500-2-251)

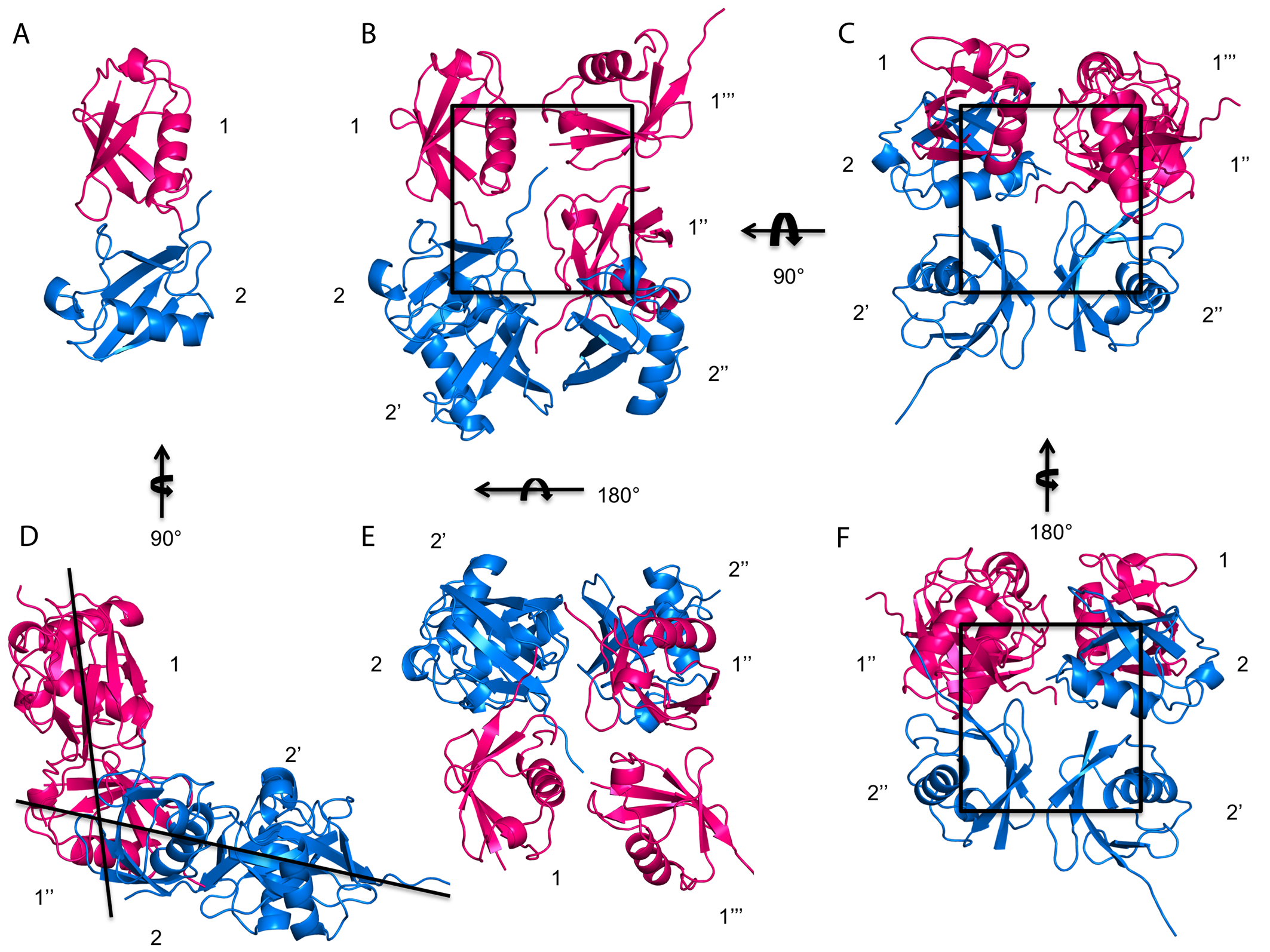

Supplement: Additional file 1 — Figure S1: Packing of molecules in crystal of Ub. A) The non-crystallographic dimer using a ribbon representation. B) Nearest symmetry molecules (at 4Å) in the same orientation as A). C, D, E) rotation of B) by 90° around the horizontal axis, at 90° along Y, at 180° along X respectively. F) rotation of C) at 180° along Y. [file 1756-0500-2-251-S1.PNG]

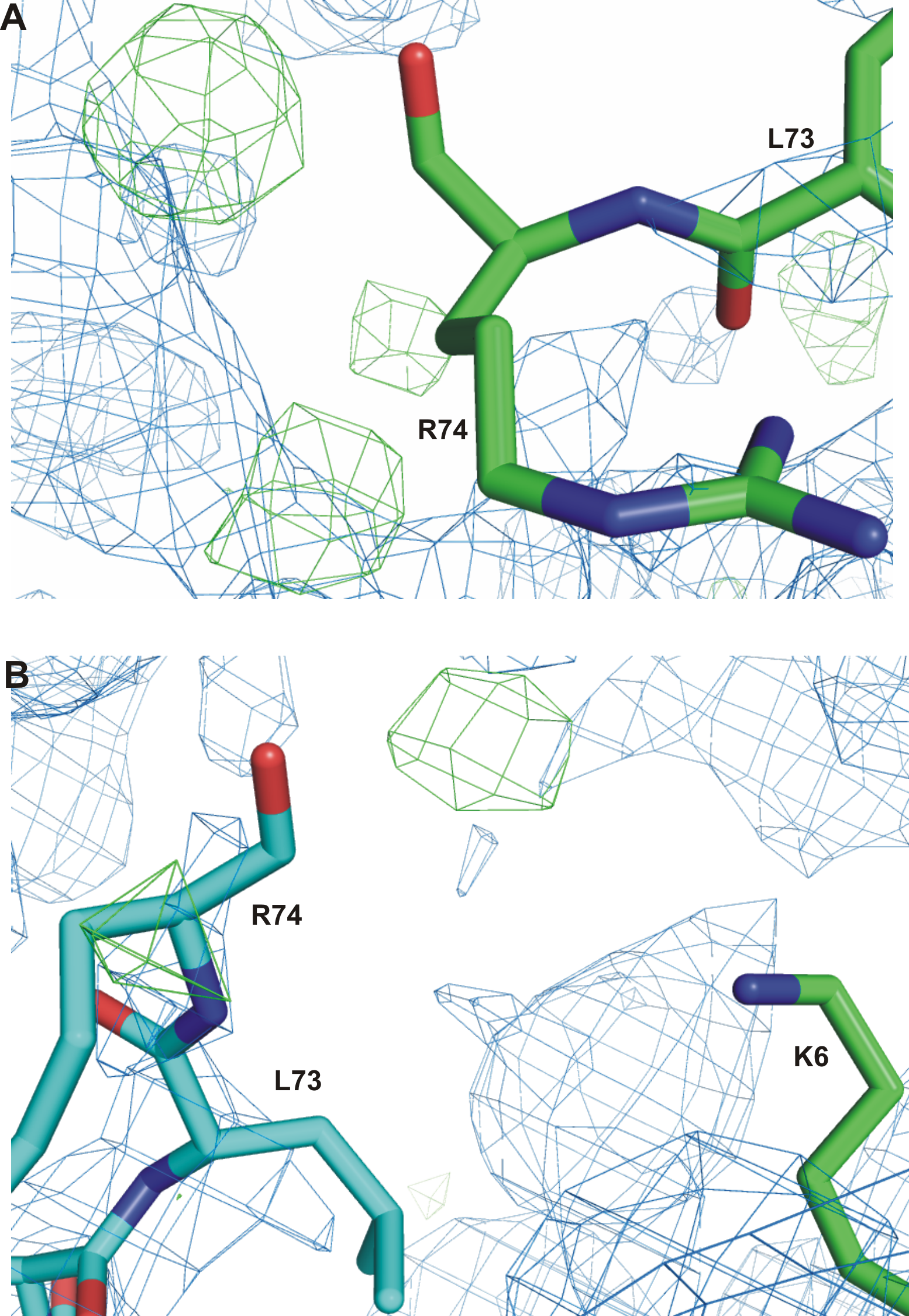

Supplement: Additional file 2 — Figure S2: Omit electron density map around the C-terminal of Ub. Omit electron density map around the C-terminal of chain A (panel A) and chain B (panel B) after removing residue Gly75 from the phase calculation. The Fo -Fc difference electron density map contoured at 3.0 σ level is coloured in green and 2Fo -Fc map contoured at 1.5 σ level (in blue). Density accounting for Gly-75 is visible for both chains. [file 1756-0500-2-251-S2.PNG]

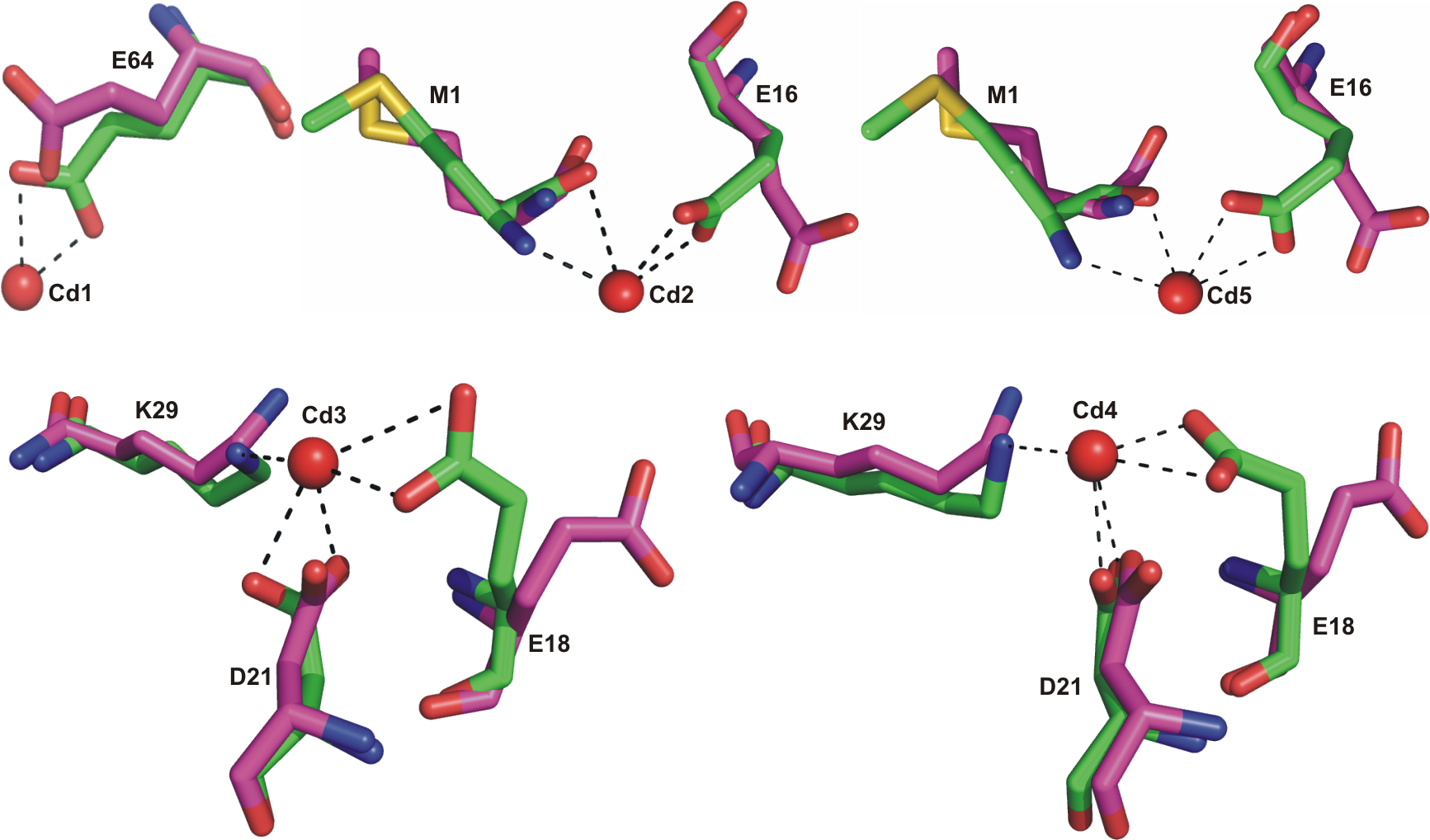

Supplement: Additional file 3 — Figure S3: Comparison between structures of native and cadmium bound Ub. Superposition of the structures of native ubiquitin (magenta) and bovine ubiquitin (green) in the region of the binding sites of cadmium (red). The interactions between cadmium ions and donor atoms are indicated by dotted lines. [file 1756-0500-2-251-S3.PNG]
